# A Wide Dynamic Range CMOS Image Sensor with a Charge Splitting Gate and Two Storage Diodes

**DOI:** 10.3390/s19132904

**Published:** 2019-06-30

**Authors:** Minho Lee, Min-Woong Seo, Juyeong Kim, Keita Yasutomi, Keiichiro Kagawa, Jang-Kyoo Shin, Shoji Kawahito

**Affiliations:** 1 Graduate School of Science and Technology, Shizuoka University, Hamamatsu, Shizuoka 432-8011, Japan; 2 Research Institute of Electronics, Shizuoka University, Hamamatsu, Shizuoka 432-8011, Japan; 3 Kyungpook National University, Buk-gu, Daegu 702-701, Korea

**Keywords:** CMOS image sensor, storage diode, high- and low-sensitivity, charge splitting gate (SG), wide dynamic range (WDR)

## Abstract

In this paper, a wide dynamic range (WDR) CMOS image sensor (CIS) with a charge splitting gate (SG) and two storage diodes (SDs) is presented. By using single-gate on/off control with the SG, photocurrent path to the first (SD1) or second storage diodes (SD2) is switched alternatively and periodically during exposure and signal electrons generated in a photodiode (PD) are transferred to and accumulated in the SD1 or SD2. By setting a large ratio of the off-time to on-time of the SG, two different sensitivity signals, which are originated by the same photodiode, are generated and a WDR image signal is obtained. This technique has a distinct advantage on mitigating the problem of motion artifact in WDR imaging with high and low sensitivity signals and flexible dynamic control of the dynamic range. An experimental WDR CMOS image sensor with 280 (H) × 406 (V)-pixel array consisting of 14 sub-arrays, each of which have 20 (H) × 406 (V) pixels, was implemented and tested. For the SG on/off-time ratio of 30 and 279, the DR of 93 dB and 104 dB, respectively, was demonstrated. The effect of the proposed WDR imaging operation on the reduced motion artifact was experimentally confirmed.

## 1. Introduction

Wide dynamic range (WDR) image sensors are recently required for a variety of applications including security systems and automobiles. Many techniques for extending their dynamic range based on CMOS image sensor (CIS) technology have been reported [1,2,3,4,5,6,7,8,9,10,11,12]. Toward the goal for developing practically-advantageous WDR image sensors, the WDR technique used in the designed image sensors should maintain important imaging quality factors and functions such as low noise, high sensitivity, high spatial resolution, less motion artifact, wide linear response and flexible control of the dynamic range below its attainable dynamic range. The LOFIC (lateral overflow integration capacitor) [7,8,9] is one of the well-performing and -balanced techniques for WDR image sensors. The possible issue of the LOFIC, however, is that the attainable dynamic range is determined and fixed by the size of capacitor used in the pixel. A flexible control of the dynamic range is not realized. To obtain a very wide dynamic range in relatively small-pixel imagers, a dedicated special process technology for building a trench structure [13] is required. The dual (multiple) sampling [4,5,12] is one of the very-practical WDR techniques. It requires reading long and short accumulation signals from the pixel. Fundamental imaging performances such as low noise, high sensitivity and good linearity are maintained and the dynamic range control is also very flexible. A possible problem of the dual sampling technique depending on its application is a motion artifact because of the time difference of capturing the long and short accumulation signals [11]. 

This paper proposes a new technique for WDR image sensors with sufficient imaging quality factors, a function of flexible control of the attainable dynamic range and mitigating the problem of motion artifact. The pixel structure is based on the standard CMOS image sensor technology with a pinned photodiode option [14]. Therefore, a low noise characteristic is self-contained. The proposed WDR pixel has two storage diodes (SDs) for storing high and low sensitivity signals, related to short and long charge accumulations. It also has a charge splitting gate (SG) for switching alternatively and periodically the photo-current flow to the two SDs. This operation is effective for mitigating the problem of motion artifact in the reproduced WDR image. The dynamic range is flexibly and dynamically controlled by the on/off time ratio of the SG. A pixel structure relevant to the proposed WDR pixel was presented by the authors [15]. However, the previous structure is not designed for alternative multiple switching of photo current to two SDs. The basic characteristics of the proposed WDR pixels were measured and the effect of the proposed operation for the reduced motion artifact in the WDR image signal was experimentally confirmed by an implemented experimental CIS chip. 

The reminder of this paper is organized as follows. Section 2 describes the principle of the proposed WDR pixel. The structure and actual design of the WDR pixel are described in Section 3. Section 4 treats CIS chip implementation and experimental results. Section 5 is for discussions on comparisons with other works and future subjects. Section 6 presents concluding remarks. 

## 2. Operation Principle of the Wide Dynamic Range Pixel

**Figure 1:**
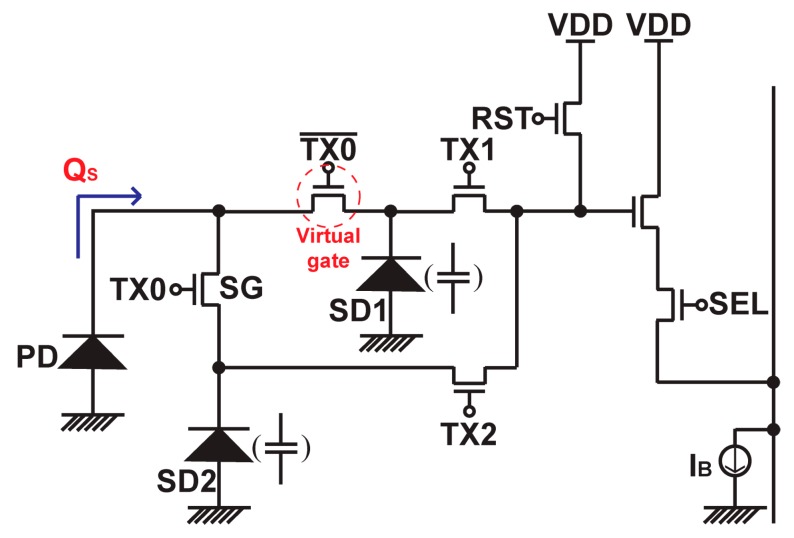
Conceptual schematic diagram of the proposed dynamic range pixel.

**Figure 2:**
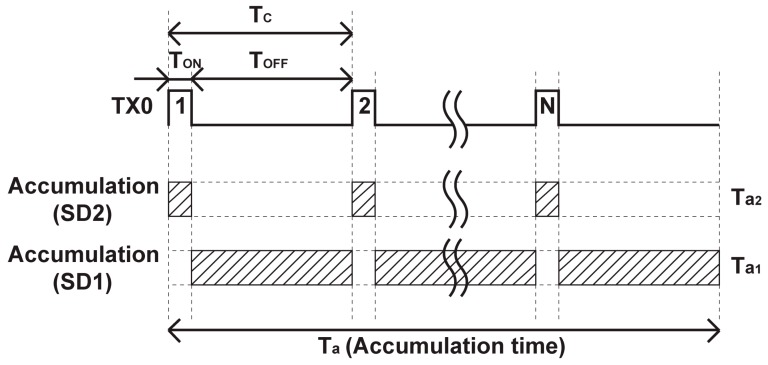
Timing diagram for accumulation of photo-charge in SD1 (C_1_) and SD2 (C_2_).

Figure 1 shows a conceptual circuit schematic diagram of the proposed wide dynamic range pixel. It has a photodiode (PD), a charge splitting gate (SG) switch transistor and two storage diodes. The storage diodes, SD1 and SD2, are connected to the PD through the SG and a virtual gate switch that works complementary to the SG. When the gate signal TX0 is set to high level, the photo-generated charge (Q_S_) flows into SD2 and the charge flowing to SD1 is stopped by the virtual gate switch. When the TX0 is set to low level, the charge flowing to the SD2 is stopped by the SG, and Q_S_ entirely flows into SD1. By turning the SG on and off periodically, the amount of charge stored in the SD1 and SD2 is controlled relatively accurately by the turn-on time of the SG if non-ideal effects such as finite switching time of the SG and light and photo-generated charge leakages are sufficiently suppressed. If this periodical switching speed in the SG is fast enough the motion artifact problem of the WDR signal reproduced by low and high sensitivity signals is mitigated. Figure 2 shows the timing diagram of accumulating photo charge in the SD1 and SD2. By periodically changing the gate signal of the SG, the accumulated photo charge ratio in the SD1 to that of the SD2 can be accurately controlled. The total photo-charge accumulation times in the SD1 and SD2, *T_a_*_1_ and *T_a_*_2_ are given by

(1) $$T_{a2}=\frac{T_{ON}}{T_{C}}\;\;T_{a}=N_{C}T_{ON}$$

and

(2) $$T_{a1}=\frac{T_{OFF}}{T_{C}}\;\;T_{a}=N_{C}T_{OFF}$$

respectively, where *T_ON_* is the gating time of the SG, or the time when TX0 is set to high level; *T_OFF_* is the time when TX0 is set to low level; *T_C_* is the cycle time of gating on and off of the SG and equals to *T_ON_* + *T_OFF_*; *N_C_* is the number of accumulation cycles; and *T_a_* is the total accumulation time. The ratio of the sensitivity *R_S_* due to signal charges in the SD1 to that of the SD2 is simply determined by the ratio of the off- to on-time given by

(3) $$Rs=\frac{T_{OFF}}{T_{ON}}$$

if there is no uncontrolled photo-signal leakage current to the SD1 or SD2. 

**Figure 3:**
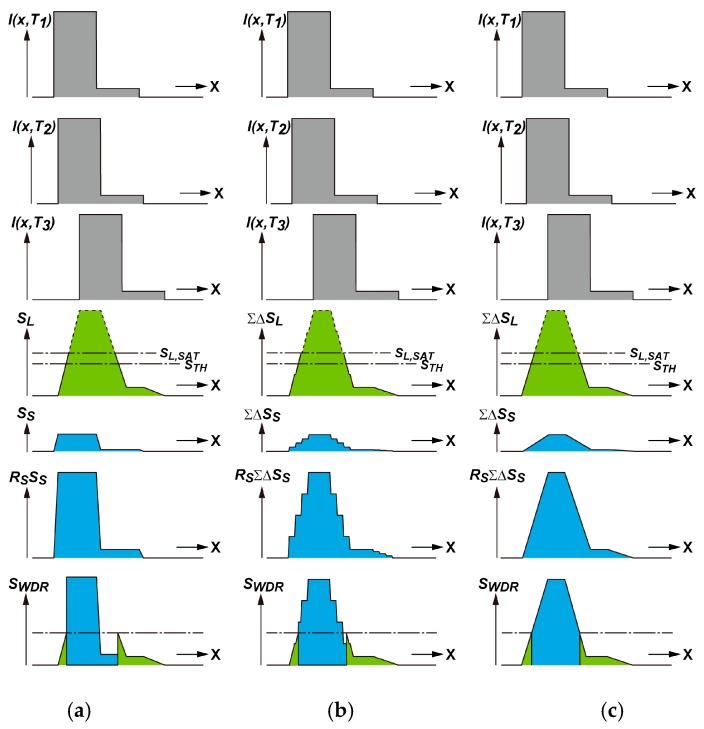
Reduction of Motion Artifact using Alternatively-Accumulated Long and Short Exposure Signals: (**a**) One Long and One Short Accumulations; (**b**) Alternative Multiple Long and Short Accumulations (Nc = 4); and (**c**) Alternative Multiple Long and Short Accumulations (Nc = infinity). *S_L,SAT_* is the saturation level of SD1 signal and *S_TH_* is the threshold level for switching from SD1 to SD2 signals.

Figure 3 shows how the alternative multiple long and short accumulations in two SDs reduce the motion artifact. It shows a one-dimensional model for simplicity. 

In Figure 3, *I(x,T*_1_*), I(x,T*_2_*)* and *I(x,T*_3_*)* are one-dimensional signal light intensity profile models of an object projected on the sensor focal plane in the coordinate of column direction *x*, at the time *T*_1_, *T*_2_ and *T*_3_, respectively, where *T*_3_ *>T*_2_ *>T*_1_. It has a unit of W/m^2^, i.e., light power per area of the focal plane. The pixel signals *S_S_(k)* and *S_L_(k)* at the column number *k*, are resulted from short-time (*T_a_*_2_) and long-time (*T_a_*_1_) photo-charge accumulations in a pixel, respectively. The unit is in volt when the signal is read out at the pixel output. The coordinate *x* takes $k\cdot p_{p}$ at the pixel center of the *k*th column, and the light intensity is assumed to be uniform within the pixel and it is given by$I(k\cdot p_{p},t)$ where *p_p_* is the pixel pitch. Since *S_S_(k)* and *S_L_(k)* are generated by the light intensity profile *I(x,t)* from *T*_1_ to *T*_2_ and from *T*_2_ to *T*_3_, respectively, they are expressed as

(4) $$S_{S}(k)=K_{S}p_{p}{}^{2}\int_{T_{1}}^{T_{2}}I(k\cdot p_{p},t)dt$$

(5) $$S_{L}(k)=K_{S}p_{p}{}^{2}\int_{T_{2}}^{T_{3}}I(k\cdot p_{p},t)dt$$

where *K_S_* is the proportionality constant. The object is supposed to be moving from left to right at a constant rate. Obviously, *T_a_*_1_ = *T*_2_ − *T*_3_ and *T_a_*_2_ = *T*_1_ − *T*_2_. In the case of one long accumulation and one short accumulation, as shown in Figure 3, the signal intensity profiles due to accumulated photo-charge signals in the SD1 and SD2 are as shown by *S_L_* and *S_S_* according to Equations (4) and (5). Although all the symbols for photo-charge signals in Figure 3 are a function of the pixel column number *k*, they are expressed as a function of the pixel column coordinate *x* for the purpose of the correspondence to the light intensity profiles. Because of the motion of the object, the x-direction profile of the SD1 signal is blurred as *S_L_* while the x-direction profile of the SD2 signal is less-blurred as *S_S_*. The blurring in image if the object is moving, also called here motion blur, occurs in all the types of image sensors if the signal accumulation time is not infinitely small. Since the amplitude of *S_S_* is *R_S_* times smaller than that of *S_L_*, the amplitude of *R_S_S_S_* is equalized to *S_L_*. A synthesized linear wide dynamic range signal *S_WDR_* is obtained by the operation as

(6) $$S_{WDR}=\{\begin{array}{c} S_{L}\\ R_{S}S_{S} \end{array}\begin{array}{c} (\mathrm{if}\;S_{L}\leq S_{TH})\\ (\mathrm{if}\;S_{L}{>S}_{TH}) \end{array}$$

where *S_TH_* is the threshold level for judging if *S_L_* is saturated or not. *S_TH_* is set at a little smaller than the saturation level of the SD1, *S_L,SAT_*, corresponding to the full well capacity (FWC) of the SD1. Because of different amounts of motion blurs in *S_L_* and *R_S_ S_S_*, the synthesized WDR signal has a structured distortion, as shown in *S_WDR_* of Figure 3a. This visible structured distortion is treated as a motion artifact and it is distinguished from the motion blurs due to the finite shutter time. Figure 3b shows the behavior of WDR imaging with alternative long and short accumulations in the SD1 and SD2 for the case of *N_c_* = 4. The totally accumulated signals in the SD1 and SD2 are denoted by ΣΔ*S_L_* and ΣΔ*S_S_*, respectively, where Δ*S_L_* and Δ*S_S_* are signal amplitudes for one cycle of accumulation. Because the durations of accumulation in the SD1 and SD2 are similar, the motion blurs of the ΣΔ*S_L_* and ΣΔ*S_S_* are also similar. Then, the one-dimensional profile of the synthesized WDR signal *S_WDR_* of Figure 3b has less structured distortion (motion artifact) than that in Figure 3a. Figure 3c shows the behavior of WDR imaging with alternative long and short accumulations in the SD1 and SD2 for the case of *N_c_* = infinity. Obviously, in this extreme case of alternative long and short accumulations in two SDs, the motion blurs of the ΣΔ*S_L_* and ΣΔ*S_S_* are the same and the WDR image signal *S_WDR_* of Figure 3c also has the same shape as that of ΣΔ*S_L_* or ΣΔ*S_S_*. 

**Figure 4:**
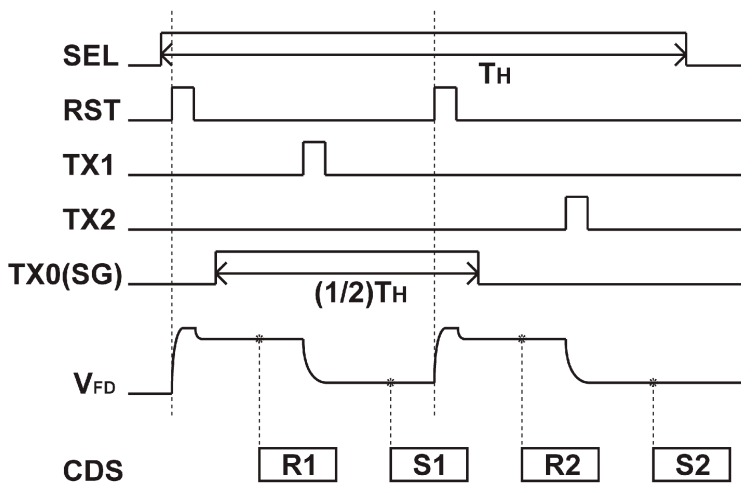
Timing diagram for reading SD_1_ and SD_2_ signals in one horizontal readout cycle.

The timing diagram for reading the two signals in the SD1 and SD2 is shown in Figure 4. The reading of two SD signals is done with a sequential charge transfer from the SD1 and SD2 to a same floating diffusion (FD) node as a true CDS (correlated double sampling) manner. The true CDS readouts for the SD1 and SD2 are done by taking the difference (*S*_1_-*R*_1_) of the first sampled reset level *R*_1_ and the first sampled signal level *S*_1_ and the difference (*S*_2_-*R*_2_) of the second sampled reset level *R*_2_ and the second sampled signal level *S*_2_, respectively. Because of the sharing of a common FD for two SD signals, the conversion gain to two SD signals is equalized. 

The WDR signal synthesis in each pixel is done by post processing. If a linear WDR signal is necessary, then the gain of *R_S_* is applied to the low-sensitivity signal and a linear WDR signal is constructed using Equation (6). If a nonlinearly compressed WDR signal is necessary for displaying the WDR image, the gain applied to the low-sensitivity signal can be set to unity, as described in Section 4. There are many algorithms for displaying or printing the WDR image using multiple different-sensitivity signals in each pixel [16]. Post-processing is useful for flexibly applying algorithms for WDR image syntheses. 

## 3. Pixel Design 

**Figure 5:**
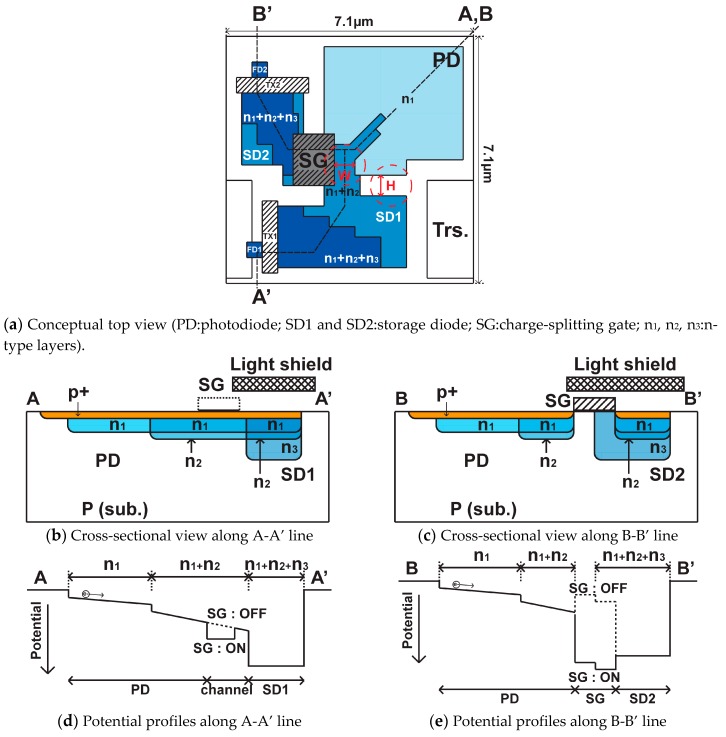
Conceptual structure and potential profiles of the WDR pixel.

Figure 5a shows the structure and layout (top view) of the proposed WDR pixel. The WDR pixel including circuits is designed to be included in the size of 7.1 μm × 7.1 μm with a 0.11 μm CIS technology. The pixel is designed such that the photo generated signal electrons in the photodiode (PD) is automatically transferred to the SD1 or SD2 according to the potential profiles made by three kinds of n-type layers and the potential control with the charge splitting gate (SG). The PD is made by two n-type doping regions with n_1_ and n_2_ layers to create a built-in drift field to transport the photo-carriers from n_1_ region to n_1_ + n_2_ channel regions. The SG is located along the n_1_ + n_2_ channel and controls the photo carriers flowing to the SD1 and SD2. The cross sections along A-A’ and B-B’ lines in Figure 5a are shown in Figure 5b,c, respectively, and the conceptual potential distributions of the route from the PD to SD1 (A-A’) and from the PD to SD2 (B-B’) are shown in Figure 5d,e, respectively. To focus only on the operation of charge transfer from the PD to SD1 or SD2, the structures and corresponding potential profiles regarding the transfer gates (TX1 and TX2) and the floating diffusions (FD1 and FD2) are not shown in Figure 5b–e. When the gate voltage of the SG is low, the SG is turned off and the potential profile in the n_1_ + n_2_ channel becomes as shown by the dashed line in Figure 5d,e. Then, the photo-generated electrons are transferred to the SD1 by the built-in drift field in the n_1_ + n_2_ channel. When the gate voltage of the SG is high, the SG is turned on and a potential dip, as shown by the solid-line in Figure 5d, is created in the n_1_ + n_2_ channel. This potential dip captures photo-electrons and disturbs the transportation of them to the SD1. The captured photo-electrons in the potential dip is once transferred to the channel region of the SG and then moved to the SD2 when the SG is turned off again because of the stepwise potential of the channel region of the SG. The potential-well depth of the SD1 or SD2 is controlled by the third n-type doping layer n_3_. The areas for the SD1 or SD2 are light-shielded with metal layers for making a large sensitivity ratio between the SD1 and SD2 and for better controllability of the sensitivity ratio by the turn-on time of the SG. 

**Figure 6:**
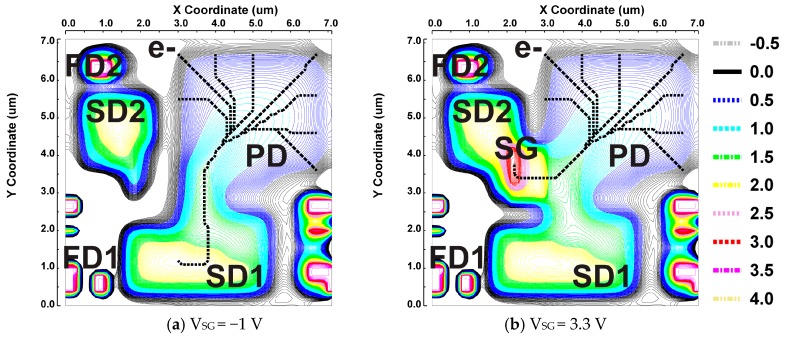
Simulated 2-D potential plots for showing the operation of the charge splitting gate.

In the actual pixel layout patterns, the n_2_ doping layer is extended to the center of the PD for creating a distinct built-in drift field to transfer all the photo-electrons to the site close to the SG gate, and the n_3_ doping layer is shaped for making the perfect charge transfer from the SD1 and SD2 to the floating diffusion nodes, FD1 and FD2, respectively, easier. Another important design issue of this pixel is the charge splitting capability by the SG gate. Ideally, the SG and the structure near the SG gate must be designed such that all the photo-electrons created in the PD are transferred to the SD2 when the SG is turned on and to the SD1 when the SG is turned off. Figure 6 shows simulated 2-D potential plots of the pixel for the SG turned off (Figure 6a) and the SG turned on (Figure 6b). The gate voltages of the SG are 3.3 V and −1 V for turning on and turning off, respectively. A device simulator, SPECTRA [17] (Link Research Corp.), was used for these and the following simulations. These 2D potential plots show the maximum potential in the depth (z-axis) range from 0 to 3 μm. Electron paths initiated by eight surrounding points of the photodiode are also shown when the SG is −1 V and when the SG is 3.3 V. This helps to understand that photo-electrons generated in the photo-diode area are mostly once gathered to the center of photodiode and then transferred to the final destination, that is, the inside of the SD1 with the SG of −1 V and the inside of the SG with the SG voltage of 3.3 V. 

A problem of the charge overflow from the SD1 to the SD2 may happen if a sufficient potential barrier is not created at the edge of the SD1 near the SG gate. This potential barrier must be large enough, even though the SG is turned on and a large amount of charge is accumulated in the SD1. The width and height of the channel region near the SG gate, which are indicated by “*W*” and “*H*” in Figure 5a, are important for realizing the charge splitting capability by the SG gate and for creating a potential barrier to prevent the overflow from the SD1 to the SD2 when the SG is turned on. The “*W*” and “*H*” of the pixel used in the design shown in Figure 5a are 0.9 μm and 1.0 μm, respectively. 

**Figure 7:**
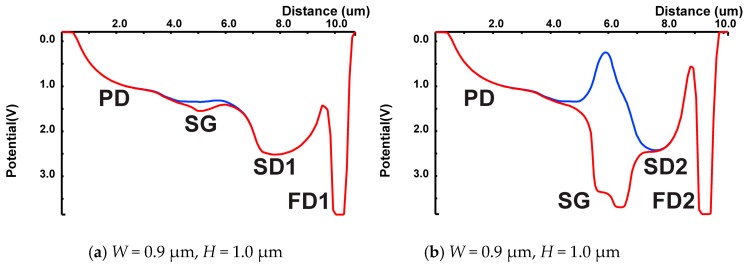
Simulated 1-D potential plots of the cross-section including those of PD, SG, SD and FD: (**a**) potential at A-A’ line (Blue: SG off, Red: SG on); and (**b**) potential on B-B’ line (Blue: SG off, Red: SG on).

Figure 7a,b shows the 1-D potential plots corresponding to the cross-sections along A-A’ and B-B’ lines in Figure 7, respectively, for the SG on (red curve) and the SG off (blue curve). When the SG is turned on, a small potential dip is created near the SG gate in A-A’ line potential profile so that photo-electrons from the PD are dropped in the dip, and the potential profile of the B-B’ line attracts the electrons in the potential dip to be transferred to the channel under the SG gate. When the SG is turned off, the electrons in the channel under the SG gate is transferred to the SD2, and photo-electrons from the PD are blocked by the large potential barrier in the B-B’ potential profile for not transferring to the SD2 and are transferred to the SD1. To prevent the charge overflow from the SD1 to SD2, the off-gate voltage of the TX1 is set to relatively high voltage of 0.5 V during exposure. The full-well capacity of the SD1 and SD2 are 6,600 e- and 8,400 e-, respectively. 

**Figure 8:**
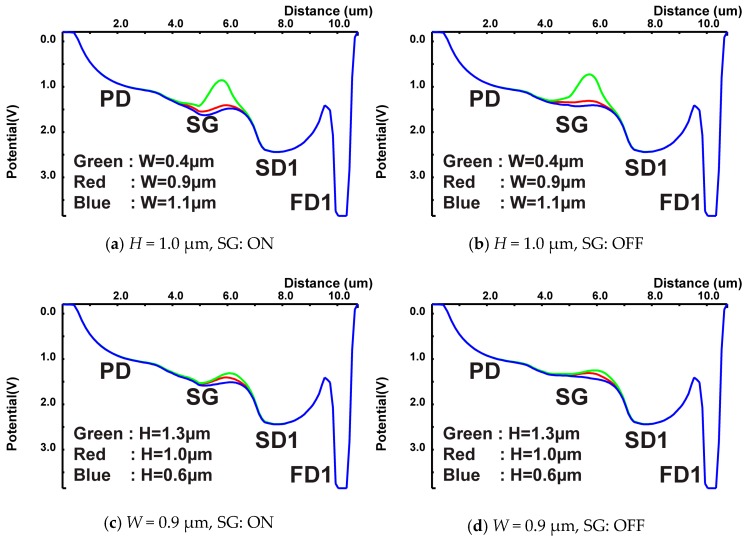
Simulated 1-D potential plots of the cross-section with variations of “*H*” and “*W*”.

Figure 8 shows simulated 1-D potential plots of the cross-sections along A-A’ line in Figure 5a, but with variations of “*H”* and “*W”*. Figure 8a,b shows the potential plots for the SG on and off, respectively, for a fixed “*H*” of 1.0 μm and “*W*” of 0.4 μm, 0.9 μm and 1.1 μm. For the “*W”* of 0.4 μm, a large potential barrier uncontrollable by the SG is created between the PD and SD1. The “*W”* of 0.9 μm or 1.1 μm is good for controlling the photo-charge flow to the SD1 or SD2, but the “*W”* of 0.9 μm is a little better for the channel potential height for preventing charge in the SD1 spilling over to the SD2. Figure 8c,d shows the potential plots for the SG on and off, respectively, for a fixed “*W*” of 0.9 μm and “*H*” of 0.6 μm, 1.0 μm and 1.3 μm. The “*H”* of 0.6 μm does not have enough potential height for preventing charge in the SD1 spilling over to the SD2 when the SG is on. The “*H”* of 1.3 μm has a small potential barrier to the SD1 in the channel. The “*H”* of 1.0 μm is a well-balanced choice for the potential barrier to the SD1 and the potential height of the channel for preventing the charge spillover from the SD1 to SD2. Since the modeling for these simulations is not perfectly accurate, several designs with different dimensions of “*W*” and “*H*” were implemented in a test CIS chip. The results of implementations with variations of “*W*” and “*H*” are discussed in Section 4. 

## 4. Implementation and Experimental Results 

**Figure 9:**
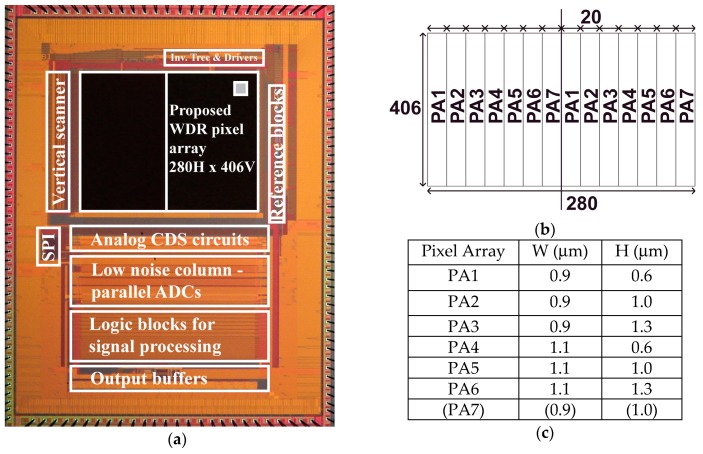
Chip layout with proposed WDR pixel structure using two SDs, test pattern arrangement with various patterns. (**a**) CIS chip photograph; (**b**) pixel arrays; (**c**) “W” and “H” of each design.

An experimental CIS chip for testing the proposed WDR pixels was implemented with a 0.11-µm CIS technology. Figure 9a shows a CIS chip photograph with the proposed WDR pixel arrays using two storage diodes. For reading image signals from the WDR pixel with low noise and wide dynamic range characteristics, multiple-sampling-based column parallel 17-bit ADC was used [18]. To obtain 17-bit noise-cancelled digital signal, the reset and photo-signal levels of the pixel output were sampled 32 times for the multiple-sampling-based A/D conversion, which is also called a folding-integration A/D conversion (FI-ADC). The FI-ADC produced 5-bit MSB (more significant bits) digital codes and an amplified analog residue, which was converted to 13-bit LSB (less significant bits) digital codes using a cyclic-based A/D conversion. The final 17-bit digital code was produced by combining MSB (5bits) and LSB (13bits) codes (=5 + 13 – 1 = 17 bits) and taking the difference of those for reset and photo-signal levels. This operation is equivalent to performing a CMS (correlated multiple sampling) in digital domain and therefore the pixel source follower’s thermal and 1/f noises were effectively reduced, while canceling reset noise and fixed pattern noise.

To experimentally find the good design for solving the problem of charge spillover from the SD1 to SD2, seven different pixel arrays whose dimensions of “*W*” and “*H*” are as shown in Figure 9b,c were included. Each pixel array, named PA1–PA7, has 406 vertical and 20 horizontal pixels. The pixel size is 7.1 µm × 7.1 µm. 

**Figure 10:**
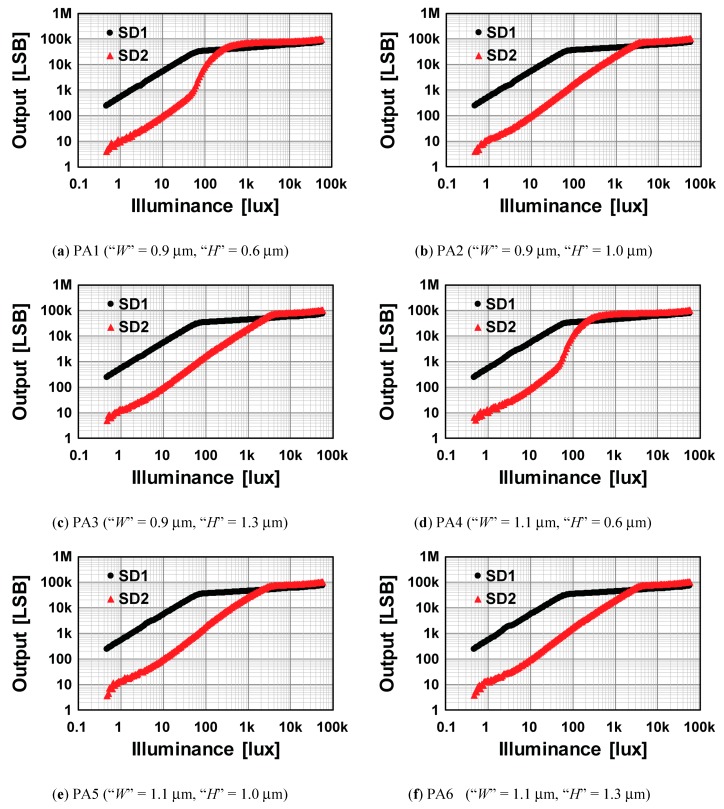
Linearity characteristics of six pixel sub-arrays.

The photo-response characteristics of the SD1 and SD2 signals for six pixel arrays (PA1–PA6) are shown in Figure 10. These curves were measured by white light with a light source box (Kyoritsu, LB-8623). The gate voltages of the SG used for this and the following measurements were 3.3 V and −1 V for turning on and turning off, respectively. The setting of *R_S_* (the ratio of *T_ON_* to *T_OFF_*) was 30. With this setting, the sensitivity ratio of the SD1 signal to that of the SD2 was 30. In these six curves, PA 1 and PA4 had a nonlinear response in the SD2 signal when the SD1 signal was saturated. This indicates that the charge spillover occurred in these two designs because PA1 and PA4 had the relatively small “*H*” of 0.6 μm compared to other designs with the “*H*” of 1.0 μm or 1.3 μm. This tendency agrees with the simulation results in Figure 9. Although the design with “H” of 1.3 μm (PA3) had a small potential barrier between the PD and SD1, a distinct difference from that with “H” of 1.0 μm (PA2) was not observed in the linearity measurements. 

As shown in Figure 7 and Figure 8, the potential in the channel of the TX1 when it is turned off is set quite close to but a little higher than the potential of the channel near the SG to prevent crosstalk from the SD1 to SD2 while maximizing full well capacity of the SD1. To carefully check the crosstalk from the SD1 to SD2, the photo-response curves of the SD2 signal for the six pixel arrays are plotted by linear-scale and the nonlinearity errors were measured. The illumination range used for the nonlinearity measurement was 0–1920 lux, and the maximum error was calculated as percent to the full-scale signal range corresponding to the illumination range. In PA1 and PA4, the nonlinearity errors were 48.0% and 55.0%, respectively. These large errors can also be seen in Figure 10. In PA2, PA3, PA5 and PA6, the nonlinearity errors were 1.06%, 0.79%, 1.26% and 0.84%, respectively. PA5 (“*W*” = 1.1 μm, “*H*” = 1.0 μm) had relatively large nonlinearity because of a higher channel potential near the SG. Since pixel arrays PA2 and PA3 had relatively good linearity and were adjacent each other at the focal plane, these two designs were mainly used for the following characterization and imaging test of the sensor. 

**Figure 11:**
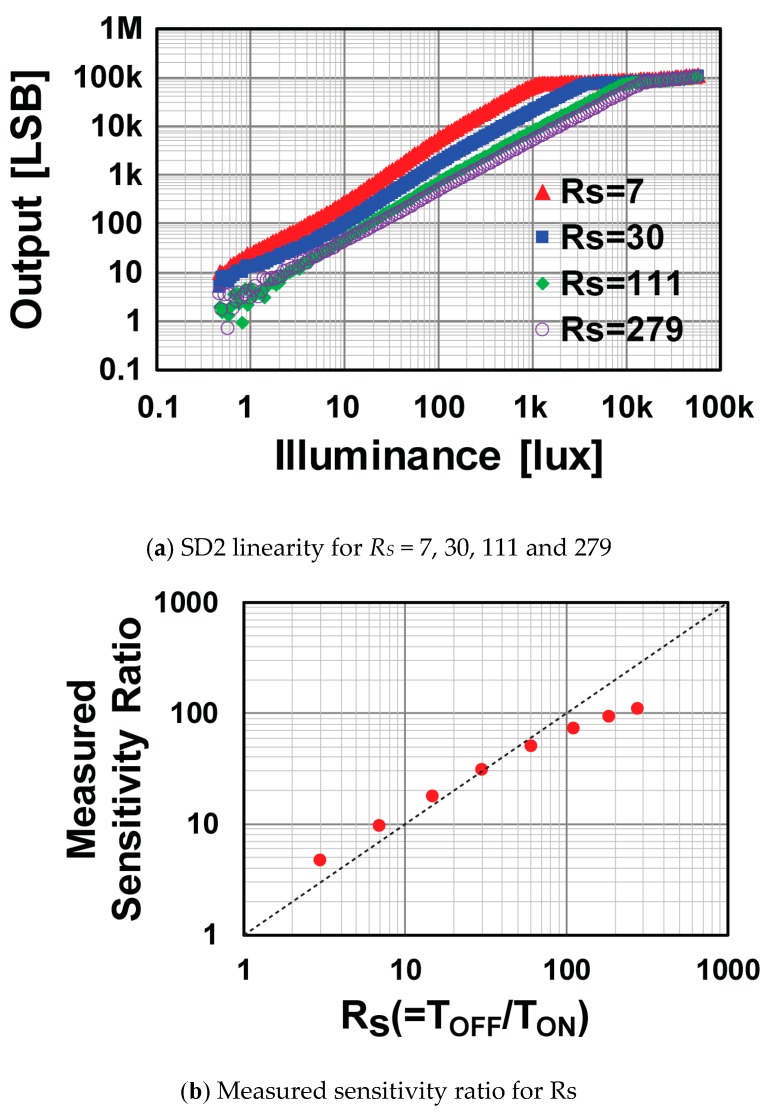
Measured photo-response characteristics for SD2 linearity and sensitivity ratio.

Figure 11a shows linearity characteristics of the SD2 signal for various on/off-time ratios *R_s_* of the SG. This ratio is controlled by the number of SG pulses as shown in the timing diagram in Figure 4. The setting of *R_S_* (the ratio of *T_ON_* to *T_OFF_*) was changed to 7, 30, 111 and 279. The sensitivity of the SD2 signal was controlled largely by the setting of *R_s_*. Figure 11b shows the relationship between the measured sensitivity ratio and the settings of *R_s_* (the ratio of *T_ON_* to *T_OFF_*). The sensitivity ratio was measured by the ratio of the output level of the SD1 to that of the SD2 at the illuminance level of 64 lux, i.e. 85% of the illumination level (= 75 lux) at which the SD1 signal was saturated. In Figure 11b, the dashed line shows the ideal case that the actual sensitivity ratio of the implemented WDR image sensor was the same as *R_S_* (the ratio of *T_ON_* to *T_OFF_*), which means the sensitivity ratio was accurately set. This was our goal. The measured sensitivity ratio was relatively accurate at *R_s_* of around 30. For small *R_s_*, the measured sensitivity ratio was larger than *R_s_*, and, for large *R_s_*, the measured sensitivity ratio was smaller than *R_s_* and was limited to 107 even if the *R_s_* was set to 279. This is because there were light and charge leakages to the SD1 and SD2 from the photodiode.

**Figure 12:**
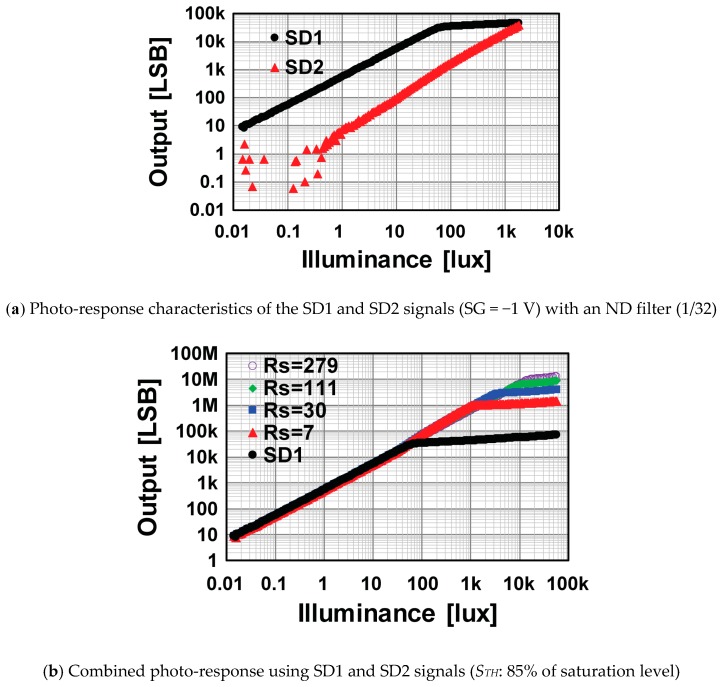
Measured photo-response characteristics including low light levels and combined photo-response.

Figure 12a shows the measured linearity characteristics for the SD1 and SD2 signals for the *R_s_* of 30. To find the linearity of the SD1 signal at the noise level, a Neutral-Density (ND) filter with the attenuation ratio of 1/32 was used. The measured temporal noise in the SD1 signal was 3.2e^−^. With this noise level, the minimum illuminance level at which the SNR was 1 (0 dB) was 0.07 lux. The saturation level of the SD2 signal was 3 klux Therefore, the dynamic range with this setting was 92.6 dB. Figure 12b shows linearity plots for the combination of the SD1 and SD2 signals using Equation (4). The lowest sensitivity ratio of the SD2 to the SD1 signals was 1/107 for the *R_s_* of 279. With this setting of the SD2 sensitivity, the saturation level of the SD2 signal was 14 klux, and the attainable maximum dynamic range is as 104 dB. 

Table 1 shows the performance summary of the implemented CIS chip. 

To evaluate the effect of the alternative multiple long and short accumulation technique for reduced motion-artifact WDR imaging, a wide dynamic range scene with a high-speed moving object was captured with the implemented CIS chip.

**Figure 13:**
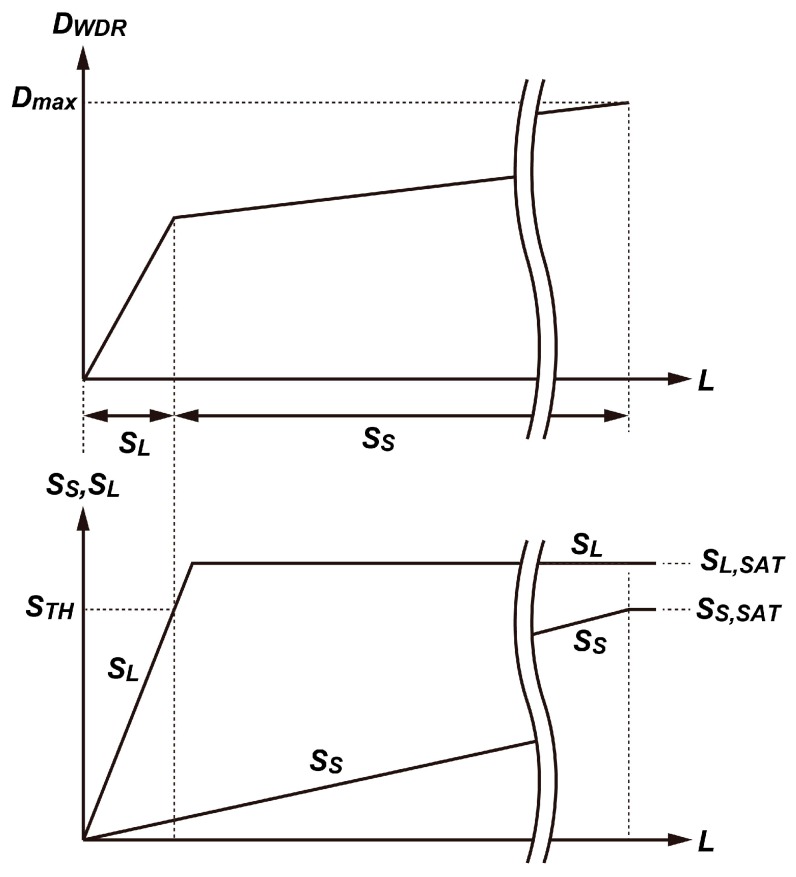
Graphical expression of Equation (7) (relationship between D_WDR_ and illuminance (L) together with the curves for *S_L_* and *S_S_* versus L).

**Figure 14:**
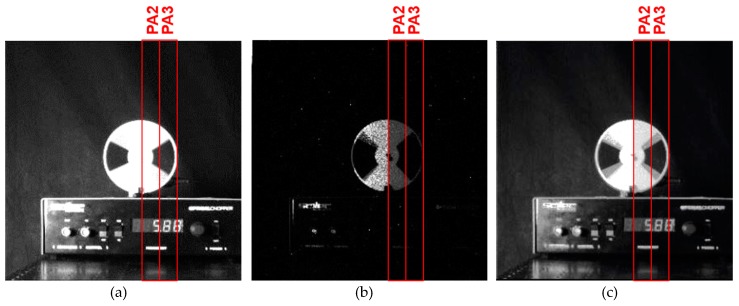
Captured images in moving object (Conventional dual sampling method): (**a**) one-time long exposure (SD1); (**b**) one-time short exposure (SD2); and (**c**) reproduced WDR image. (*S_TH_*: 85% of saturation level).

The implemented experimental CIS chip contained seven kinds of pixel sub-arrays, as shown in Figure 9, and the sub-arrays PA2, PA3, PA5 and PA6 had good linearity, as shown in Figure 10. In the following imaging experiments, PA2 and PA3 were used for the evaluation of the motion artifact, although the image is taken by the whole array. A wide dynamic range signal for displaying images *D_WDR_* was obtained by using a long accumulation signal *S_L_* (SD1 signal) and short accumulation signal *S_S_* (SD2 signal) and with a simple piecewise-linear curve synthesis expressed as

(7) $$D_{WDR}=\{\begin{array}{cc} D_{\max}\frac{S_{L}}{S_{TH}(1-R_{S}{}^{-1})+S_{S,SAT}} & (\mathrm{if}\;S_{L}\leq S_{TH})\\ D_{\max}\frac{(1-R_{S}{}^{-1})S_{TH}+S_{S}}{S_{TH}(1-R_{S}{}^{-1})+S_{S,SAT}} & (\mathrm{if}\;S_{L}{>S}_{TH}) \end{array}$$

where *D_max_* is the maximum digital number for displaying and *S_S,SAT_* is the saturation signal level of the SD2 signal. Equation (7) is graphically expressed in Figure 13. In the following imaging tests, *S_TH_* was set at 85% of the saturation signal level *S_L,SAT_*. In Equation (7), if the signal amplitude of *S_L_* is smaller than or equals to *S_TH_*, *S_L_* is used for producing *D_WDR_* and if the signal amplitude of *S_L_* is larger than *S_TH_*, *S_S_* is used for producing *D_WDR_*. If *S_L_* = 0, *D_WDR_* takes 0 and for *S_S_* = *S_S,SAT_*, *D_WDR_* takes *D_max_*. If *S_L_* is exactly same as *S_TH_*, *D_WDR_* takes $D_{WDR}=D_{\max}S_{TH}/(S_{TH}(1-R_{S}{}^{-1})+S_{S,SAT})$. If *R_S_* = 30, *S_TH_* = 0.85*S_L,SAT_* and *S_S,SAT_* = *S_L,SAT_*, *D_WDR_* nearly equals to 0.47*D_max_*. With this setting, the knee point of the piecewise linear WDR curve was set at 47% of *D_max_*. Images in Figure 14 were captured with the implemented chip, but the signal accumulation in the SD1 and SD2 was done by the conventional operation, i.e., the SD1 signal was acquired by one-time long accumulation of 31.2 ms, and, after that, the SD2 signal was acquired by one short-time accumulation of 1.06 ms. In the scene, a chopper blade was rotating at 180 rpm. The background in the scene was very dark and the white chopper blade was very bright, which were captured by *S_L_* (SD1 signal) and short accumulation signal *S_S_* (SD2 signal). As shown in Figure 14, a motion artifact at around the edge of the chopper blade was observed. 

**Figure 15:**
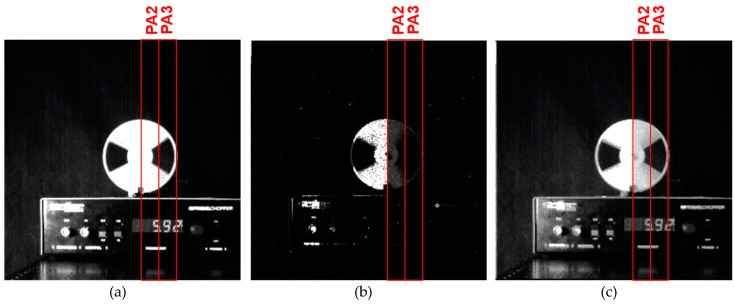
Captured images in moving object (proposed multiple sampling method): (**a**) multiple-time long exposure (SD1); (**b**) multiple-time short exposure (SD2); and (**c**) reproduced WDR image. (*S_TH_*: 85% of saturation level).

Figure 15 shows the images taken with the proposed alternative multiple long and short signal accumulations. One SG-on time was 57.5 μs and it was repeated 18 times. With this setting, the total accumulation time for the SD2 signal was 1.035 ms. One SG-off time for signal accumulation in the SD1 was set to 1782.5 μs (=57.5 μs × 31) and it was repeated 17 times alternatively with accumulating for the SD2 (SG: on). Including the extra 862.5 μs, the total accumulation time for the SD1 signal was 31.165 ms. *R_S_*, the ratio of sensitivity being 31.165/1.035≅30.

Because of the duration of signal accumulations, in each pixel for SD1 and SD2 signals were almost the same using the alternative multiple long and short signal accumulation technique. Natural motion blur was observed only at around the edge of the chopper blade, indicating the effectiveness of the proposed WDR pixel for the reduced motion artifact.

## 5. Discussions

In this section, a comparison of the proposed wide dynamic range CMOS image sensors with other technologies and the possible future works are discussed. 

In Table 2, a comparison of the proposed WDR technique with two typical and well-established WDR Techniques is shown. Dual sampling [1] and multiple sampling [12] are practical and advantageous techniques for extending the dynamic range. The dynamic range is controlled very flexibly and in very wide range by reading two different accumulation-time signals from the same pixel in one frame. Attainable dynamic range is also very high and it is relatively easily applicable to small pixel because no modification to pixels is necessary. The possible issue is the motion artifact when the object is moving because of the difference of the charge accumulation timing of the two signals. The WDR image sensor with LOFIC (lateral overflow integration capacitor) pixels is another practical technique for extending the dynamic range. The important advantage is the good SNR at high-illumination region because signal charges are linearly accumulated with a large-size capacitor. Motion artifact is also small because it uses seamless linear integration of photo-generated electrons from dark to bright scene. A possible issue is the small pixel applicability and the attainable dynamic range with the small-size pixel. This is because the dynamic range is limited by the capacitor size used in each pixel. The proposed technique using two storage diodes (capacitors) with different accumulation time ratio but almost the same duration using alternative multiple-time charge transfer is a well-balanced technique. The problem of motion artifact in the WDR image sensors with two accumulation-time signals is solved. The dynamic range can be flexibly and widely controlled, and the attainable dynamic range is also sufficiently high.

Since this paper reports a basic study on WDR image sensors with two storage diodes (SDs) whose charge accumulations are flexibly controlled, there are many issues to be improved toward a higher-performance WDR image sensor with practical merits. Currently, the full-well capacity of the SD1 is limited to 3500 e^−^ with the size of 3.9 × 1.8 μm^2^, that is 500 e^−^/μm^2^, which is quite small compared with the charge density of pinned photodiodes of standard CMOS image sensor technology, where more than 5000 e^−^/μm^2^ can be realized depending on the process technology used. The noise level achieved was also not surprisingly small when compared with CIS chips with the same column ADC technology [19], where the read noise level below 1 e^−^ has been realized. With improvements of the full well capacity and the read noise, a proposed WDR image sensor with the dynamic range of more than 120 dB, for example, will be realized. 

Presently, the measured sensitivity ratio is different from *R_S_* (the ratio of the turn-off to turn-on time of the SG) particularly if *R_S_* is set to a very-large ratio. The reduction of the photo and charge leakages in the storage diodes for better controllability of the sensitivity ratio of the SD1 to SD2 signals is another important issue for a better pixel photo response non-uniformity (PRNU) and the reduction of pixie-to-pixel deviation of the nonlinearity at switching point of the SD1 and SD2 in the synthesized WDR signals. The optimization of microlens, light shielding structures, and photo-charge shielding structures for photo-electrons generated in the deep inside of silicon will be necessary for better light and charge shielding in the SD1 and SD2. To do this, more advanced simulations with combined optical/electrical simulation tools would be useful. These are left as a future work. 

The effectiveness of the technique proposed for reducing the motion artifact in the synthesized WDR image was not quantitatively evaluated. To do this, we need to build a model for quantitatively calculating the amount of motion artifacts, define quantitative parameters and specify conditions for evaluating the motion artifacts such as the speed of moving object, imager’s readout speed, type of shutters (global or rolling), either moving picture or still image and consideration of human perception. These are important for the next stage of study with adaptive dynamic control for minimizing visible motion artifact, maximizing the SNR and optimizing the dynamic range to the scene. 

## 6. Conclusions

A wide dynamic range (WDR) CMOS image sensor (CIS) using two storage diodes (SDs) and a charge splitting gate (SG) is proposed and a proof-of-concept CIS chip was implemented and evaluated. By splitting the signal electrons from the pinned photodiode (PPD) to two storage diodes (SD1 and SD2), the dynamic range (DR) of the image sensor is flexibly and accurately controlled by the on/off ratio of the SG. Using the alternative multiple long and short accumulations in two storage diodes in each pixel, a reproduced WDR image with reduced motion artifact and the dynamic range of larger than 90 dB is realized. For wider dynamic range, better image quality and more accurate dynamic-range control, increase of the full-well capacity in the storage diodes and reduction of light and charge leakage will be necessary. 

## Figures and Tables

**Table 1:** Chip Characteristics.

**Process**	0.11-µm CIS with pinned photodiode option
**Pixel Size**	7.1 µm × 7.1 µm
**Pixel Count**	280 (H) × 406 (V)
**Fill Factor**	27.31%
**ADC Resolution**	17 bit
**Conversion Gain**	76.2 µV/e^−^
**Sensitivity**	56 Ke^−^/lux·s (2850 K)
**Noise**	3.2 e^−^rms (@median)
**Frame Rate**	30.9 fps
**Dynamic Range**	60 dB (@ SD1) 63 dB (@ SD2) 93 dB (@ *R_S_ = 30*) 104 dB (@ *R_S_ = 279*)

**Table 2:** Comparison of Three Wide Dynamic Range Image Sensor Technologies.

	Multiple Sampling (Multiple Sampling at Different Timing)	LOFIC (Overflow Integration Capacitor)	This Paper (Dual Storage and Multiple Transfer)
Dynamic Range (max.)	112 dB @ 3.75 μm [12] 109 dB @ 20.45 μm [16]	102 dB @ 4.2 μm [8] 130 dB @ 16 μm * [13]	104 dB @ 7.1 μm
Motion Artifact	Large	Small	Small
Small Pixel Applicability	Very Good	Fair	Fair
Full Well Capacity (Shot-noise-limited SNR)	Good	Very Good	Fair (to be improved)
Dynamic Range Control	Flexible	Fixed	Flexible

* Dedicated process for a trench capacitor is used.
